# The complete plastid genome of *Grateloupia filicina* (Rhodophyta) and phylogenetic analysis

**DOI:** 10.1080/23802359.2018.1524279

**Published:** 2018-10-30

**Authors:** Jing Zhang, Xianming Tang, Wei Zhou Chen, Tao Liu, Yue Li

**Affiliations:** a Qilu University of Technology (Shandong Academy of Sciences), Jinan, People’s Republic of China;; b Hainan Academy of Ocean and Fisheries Sciences, Haikou, People’s Republic of China;; c Marine Biology Institute, Shantou University, Shantou, People’s Republic of China;; d College of Marine Life Sciences, Ocean University of China, Qingdao, People’s Republic of China;; e WuXi NextCODE, Shanghai, People’s Republic of China

**Keywords:** *Grateloupia filicina*, plastid genome, phylogenetic analysis

## Abstract

In this study, we obtained the complete plastid genome of *Grateloupia filicina* (Lamouroux) C. Agardh using the high-throughput sequencing method. It had circular mapping organization with the length of 195,990 bp and contained 265 genes including 195 protein-coding genes, three rRNA genes, one tmRNA gene, 28 tRNA genes and 38 unidentified open reading frames (ORFs). Moreover, phylogenetic analysis revealed that *G. filicina* firstly clustered with *Grateloupia taiwanensis.* The complete plastid genome obtained in this work would be useful for further understanding the evolution of *Grateloupia*.

Red alga *Grateloupia filicina* (Lamouroux) C. Agardh (Halymeniales, Rhodophyta) is an edible marine macroalga which is distributed globally from tropical to warm temperate regions (Wynne 1998; Masuda et al. 2000). In recent years, *G. filicina* has attracted us in a considerable attention owing to its multiple biological functions. The sulphated polysaccharide extracted from *G. filicina* has been reported to have high anticoagulant activity (Nikapitiya et al. 2007), anti-HIV-1 activity (Wang et al. 2007), antiviral activity (Sun et al. 2017) and antiangiogenic effects (Yu et al. 2012). Here, we determined the complete plastid genome of *G. filicina* (GenBank accession number: MG598531) and constructed phylogenetic analysis to provide new molecular information.

*G. filicina* samples (specimen number: 2016030029) were collected from Xiangshan Harbor, Zhejiang Province in eastern China (29°30′20′N, 121°35′6′′E) and deposited in the Culture Collection of Seaweed at the Ocean University of China. Paired-end reads were sequenced using the HiSeq × Ten system (Illumina, USA). Approximately 9 Gb of paired-end (150 bp) clean reads were randomly retrieved from the total sequencing output and used as input into NOVOPlasty (Dierckxsens et al. 2017) to assemble the plastid genome. The plastid genome of *Grateloupia taiwanensis* (GenBank accession number: KM999231) was used as the reference sequence. Transfer RNA genes were identified using the tRNAscan-SE Search Server (Schattner et al. 2005). Other regions were annotated by comparison with those of *G. taiwanensis* by using Geneious R10 (Biomatters Ltd., Auckland, New Zealand). Phylogenetic analysis based on *rbc*L and 16S genes from 17 Rhodymeniophycidae plastid genomes were conducted using Maximum likelihood (ML) method. ML tree search and ML bootstrap analysis were performed using RaxML (Stamatakis 2006). *Asparagopsis taxiformis* (NC_031148) was used as an outgroup.

The complete plastid genome of *G. filicina* was a circular DNA of 195,990 bp. The overall AT content was 69.1% exhibiting a high AT richness. The plastid genome contained a set of 265 genes including 195 protein-coding genes, three rRNA genes, one tmRNA gene, 28 tRNA genes, and 38 unidentified open reading frames (ORFs). Gene arrangement and component of its plastid genome were similar to those of *G. taiwanensis* indicating the conserved evolution. ML analyses showed that *G. filicina* firstly clustered together with *G. taiwanensis* (Figure 1). And the clustering relationship of 17 red algae supported the previous classification system. The complete plastid genome provided in this study would be useful for further understanding the evolution of *Grateloupia*.

**Figure 1.**
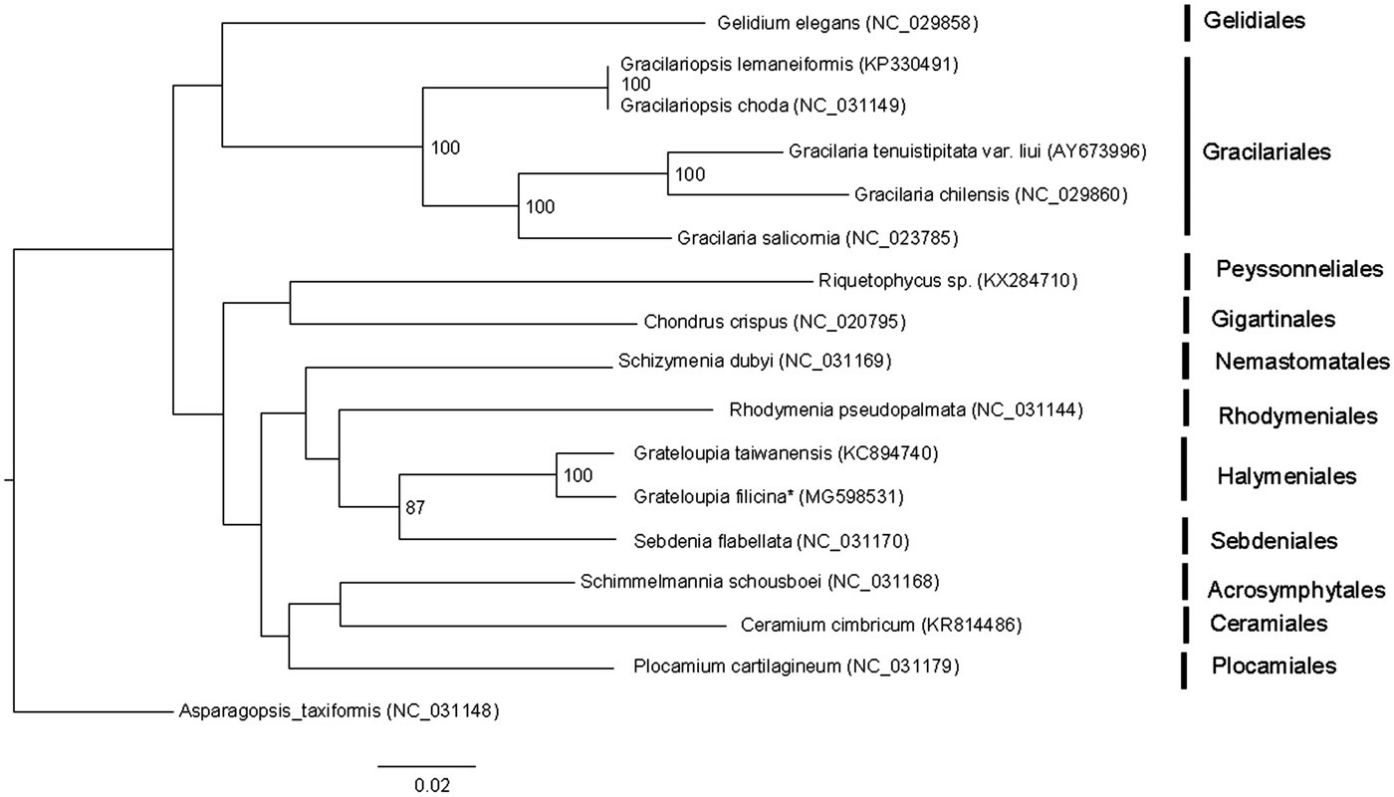
Phylogenetic tree (maximum likelihood) of 17 representative Rhodymeniophycidae species based on *rbc*L and 16S genes. Numbers along branches are RaxML bootstrap supports based on 1000 nreps (<70% support not shown). Asterisks after species names indicate newly determined mitochondrial genomes.
